# Oral Delivery of Double-Stranded RNAs and siRNAs Induces RNAi Effects in the Potato/Tomato Psyllid, *Bactericerca cockerelli*

**DOI:** 10.1371/journal.pone.0027736

**Published:** 2011-11-16

**Authors:** Hada Wuriyanghan, Cristina Rosa, Bryce W. Falk

**Affiliations:** 1 Department of Plant Pathology, University of California Davis, Davis, California, United States of America; 2 Department of Entomology, The Pennsylvania State University, University Park, Pennsylvania, United States of America; Ghent University, Belgium

## Abstract

The potato/tomato psyllid, *Bactericerca cockerelli* (*B. cockerelli*), and the Asian citrus psyllid, *Diaphorina citri* (*D. citri*), are very important plant pests, but they are also vectors of phloem-limited bacteria that are associated with two devastating plant diseases. *B. cockerelli* is the vector of *Candidatus* Liberibacter psyllaurous (solanacearum), which is associated with zebra chip disease of potatoes, and *D. citri* is the vector of *Ca*. Liberibacter asiaticus, which is associated with the Huanglongbing (citrus greening) disease that currently threatens the entire Florida citrus industry. Here we used EST sequence information from *D. citri* to identify potential targets for RNA interference in *B. cockerelli*. We targeted ubiquitously expressed and gut-abundant mRNAs via injection and oral acquisition of double-stranded RNAs and siRNAs and were able to induce mortality in recipient psyllids. We also showed knockdown of target mRNAs, and that oral acquisition resulted primarily in mRNA knockdown in the psyllid gut. Concurrent with gene knockdown was the accumulation of target specific ∼ 21 nucleotide siRNAs for an abundant mRNA for *BC-Actin*. These results showed that RNAi can be a powerful tool for gene function studies in psyllids, and give support for continued efforts for investigating RNAi approaches as possible tools for psyllid and plant disease control.

## Introduction

The potato/tomato psyllid, *Bactericerca cockerelli* (*B. cockerelli*), is a serious pest of potato and tomato in several regions of the world (http://www.eppo.org/QUARANTINE/Alert_List/bacteria/Liberibacter_psyllaurous.htm), and is associated with plant diseases including psyllid yellows of tomatoes [1] and zebra chip disease of potatoes [2]. Psyllid yellows is likely associated with feeding damage and toxin production by *B. cockerelli*, but this psyllid is also a vector of the phloem-limited bacterium, *Candidatus* Liberibacter psyllaurous (solanacearum), which is believed to be the causal agent of zebra chip disease. Zebra chip is rapidly emerging as a widespread and important disease of potatoes in many potato-growing regions worldwide, and in many ways it is very similar to another plant disease, Huanglongbing (HLB), or Citrus greening. HLB is believed to be caused by a phloem-limited bacterium, *Ca.* Liberibacter asiaticus which is transmitted from plant-to-plant by the Asian citrus psyllid, *Diaphorina citri* (*D. citri*) [3]. HLB is presently the world's most important citrus disease, causing serious losses wherever it occurs. While HLB is relatively new to the U. S., it is now widespread in Florida posing a serious threat to the entire Florida citrus industry [4]. New strategies to control *D. citri, B. cockerelli* and/or HLB and zebra chip are greatly needed. The biological similarities of HLB to zebra chip, and the phylogenetic conservation of the causal agents of the diseases and their psyllid vectors suggest that success with one insect species, *B. cockerelli*, will have potential application to the other, *D. citri*.

RNA interference (RNAi) is a natural gene regulation and antiviral defense system of eukaryotic cells. RNAi functions as a “gene silencing” mechanism by targeting specific RNA sequences for degradation (or possibly translational inhibition) resulting in eliminating or reducing expression of the RNA target [5], [6]. RNAi has rapidly become an intensely studied research area with great potential for both fundamental and practical biology. RNAi-based strategies for conferring plant resistance to bacterial, nematode and virus-induced plant diseases have been demonstrated [7]–[9], and recently have been studied for a number of insect systems [10], even leading to the suggestion of developing “insect-proof plants” [11], [12]. Whether or not RNAi strategies could be useful for controlling psyllids (and their associated plant diseases) is currently unknown, but based on results with other insects it is an important question to assess. Furthermore, while *B. cockerelli* is an important psyllid, it may also serve as a more tractable model for the very important citrus-associated *D. citri* because the former has a wide host range among solanaceous herbaceous plants (tomato, tobacco and potato).

Most insect RNAi studies have used specific double-stranded RNAs (dsRNAs) as effectors to induce RNAi activity in recipient insects[10]. The dsRNA effectors have been delivered either via intrathoracic injection or via oral feeding. Injection of dsRNAs is widely used and proven to be effective in many insect species including the pea aphid (*Acyrthosiphon pisum*), honeybee (*Apis mellifera*), flour beetle (*Tribolium castaneum*) and grasshopper (*Schistocerca Americana*) [13]–[16]. Recently, successful RNA knockdown effects via artificial feeding of dsRNAs have also been reported in insects including the Triatomine Bug (*Rhodnius Prolixus*), light brown apple moth (*Epiphyas postvittana*), tsetse fly (*Glossina morsitans morsitans*) and diamondback moth (*Plutella xylostella*) [17]–[20]. Interestingly, species-specific selective insecticidal effects of orally-delivered dsRNA for different insect species including the fruit fly (*Drosophila melanogaster*), flour beetle (*Tribolium castaneum*), pea aphid (*Acyrthosiphon pisum*) and tobacco hornworm (*Manduca sexta*) were also reported in a parallel experiment [21]. Most strikingly, RNAi activity has been induced by plant-expressed hairpin double-stranded RNAs [22]–[24]. These exciting findings highlight the possibility of using RNAi technology for future plant transgene-mediated efforts to control specific insect pest species.

In the present study, we demonstrated for the first time RNAi effects in *B. cockerelli*, as manifested by the specific downregulation of endogenous mRNA and the production of ∼21 nucleotide siRNAs, which are specific products of the RNAi pathway. Delivery of gene-specific dsRNAs or siRNAs (targeting *Actin*, *ATPase*, *Hsp70* or *CLIC*) by microinjection or by a newly developed oral delivery protocol induced RNAi effects in teneral adult *B. cockerelli* and decreased survival of recipient psyllids. Our oral delivery system will allow the efficient screening of psyllid candidate gene targets for RNAi as more sequence information (for both *B. cockerelli* and *D. citri*) becomes available.

## Materials and Methods

### 1. Insects and plants

The *B. cockerelli* used in this study were obtained from Dr. T. D. Paine's laboratory, University of California, Riverside[1]. Psyllids were reared on tomato plants (*Solanum lycopersicum* Early Pak 7) in mesh cages within growth chambers at 25°C and 70% humidity under 14∶10 (light: dark) photoperiod. Teneral adult psyllids were used for all of the experiments.

### 2. Psyllid RNA isolation, cDNA synthesis and Northern blot analysis

Total RNAs were extracted from whole *B. cockerelli* or specific tissues using the RNeasy Mini Kit (Qiagen). To isolate *B. cockerelli* gut RNAs, adult psyllids were immobilized at −20°C for 10 min, quickly transferred onto an ice box, and dissected with forceps while examining with a dissecting microscope. Approximately 100 individuals were dissected for each experiment and total gut RNA was isolated from pooled guts. Simultaneously, other tissues including head, abdomen, thorax, and reproductive organs were collected for total RNA extraction. cDNA was synthesized from 1 µg total RNAs using SuperScript® III First-Strand Synthesis System from Invitrogen (catalog NO. 11904-018).

Northern blot hybridization analysis was performed using 1.5 µg of total RNAs of *B. cockerelli* and *D. citri* (*D. citri* total RNAs were kindly provided by Dr. W. O. Dawson, University of Florida). The total RNAs were denatured using glyoxal and separated on 1% agarose gels in HEPES-EDTA buffer, then transferred to nylon membranes (Amersham Hybond™-NM) using the methods described previously [25]. Hybridization was performed using gene-specific probes in PerfectHyb™ Plus Hybridization Buffer (Sigma, catalog NO. H7033). The pGEM®-T Easy plasmids containing *BC-Actin*, *BC-ATPase, BC-Hsp70* or *BC-CLIC* sequences were digested with restriction endonucleases PstI, NcoI, SphI or SphI, respectively, and the linearized plasmids were used as templates for the labeling reaction described below. ^32^P-UTP-labeled negative strand *BC-Actin*, *BC-ATPase, BC-Hsp70* or *BC-CLIC* RNA transcripts were generated *in vitro* using T7, SP6, SP6 or SP6 RNA Polymerase (Ambion, catalog NO. AM1320) respectively and used as probes for hybridization. Hybridizations were performed at 65°C overnight. Blots were washed by shaking in low stringency wash solution [2 X SSC, 0.1% (w/v) SDS] at 25°C for 15 min, followed by medium stringency wash solution [0.5 X SSC, 0.1%(w/v) SDS] at 25°C for 15 min, and finally in high stringency wash solution [0.1 X SSC, 0.1% (w/v) SDS] at 65°C for 15 min. Blots were removed, drained, wrapped in SaranWrap and exposed to X-ray film at −80°C. The exposure times for *BC-Actin*, *BC-ATPase*, *BC-Hsp70* and *BC-CLIC* were 15 h, 2 h, 38 h and 38 h, respectively.

### 3. dsRNA and siRNA preparation

We used both dsRNAs and small interfering RNAs (siRNAs) for RNAi assays. The dsRNAs were synthesized via *in vitro* transcription using the MEGAscript RNAi kit (Ambion, catalog NO. AM1626) using PCR products as templates for each target. The T7 primer sequence was placed in front of both the forward and reverse primers which were used for PCR amplification of the template for dsRNA synthesis (Table S1). The dsRNA quality was monitored by agarose gel electrophoresis and the concentration was determined by spectrophotometry. In some cases, dsRNAs were digested with ShortCut® RNase III (New England Biolabs, catalog NO. M0245S) to prepare ∼21nt fragments (siRNAs) according to the manufacturer's protocol. Briefly, 10 µg dsRNAs were digested with ShortCut RNase III in a 100 µL reaction at 37°C for 30 min. Ten µL 10 X EDTA (0.5 M) was added to stop the reaction, and the small RNAs were precipitated with ethanol and dissolved in ddH2O.

### 4. dsRNA injection

DsRNA injection assays were performed using a micro-manipulator and an insect injection system (http://www.tritechresearch.com/MINJ-PD.html) fitted with a glass capillary needle. Psyllids were immobilized at −20°C for 10 min, transferred into a box that was placed on ice prior to injection with the dsRNAs. Each individual was injected with 200 nL of 100 ng/µL dsRNAs between the 2^nd^ and 3^rd^ abdominal segments. After injection, the psyllids were placed onto the leaves of tomato seedlings, caged and maintained in a growth chamber as described above. Mortality was scored daily, and total RNAs from individual live psyllids were extracted at two days post injection and used for cDNA preparation and quantitative real-time PCR.

### 5. Feeding assays

Feeding assays were performed using teneral adult psyllids which were allowed to feed on an artificial diet solution consisting of 15% (w:v) sucrose, contained between two layers of stretched parafilm. Feeding assays were performed at room temperature in the dark. In order to visually determine if psyllids fed on test solutions, green food coloring, fluorescent-labeled dsRNAs, or a GFP PCR product was added to the feeding solutions. Green food coloring (McCORMICK & CO) was supplied with 1/10 (v:v) dilution in artificial diet and green abdominal coloration was visualized in psyllids after 24 h feeding. Fluorescent-labeled GFP dsRNAs were prepared using a Silencer® siRNA Labeling Kit - Cy™3 (Ambion, catalog NO. AM1632) according to the manufacturer's protocol. Briefly, 10 µg dsRNA was labeled in 50 µL reaction at 37°C for 1 h, the Cy™3 –labeled dsRNA was ethanol precipitated and the free Cy™3 was removed in the supernatant. The resulting Cy™3 –labeled dsRNA was dissolved in 100 µL ddH2O. 50 ng/µL Cy™3-labeled dsRNA was supplied in artificial diet, and the fluorescence was visualized by fluorescence microscopy (Leica DM5000B) 48h after the start of feeding. The ability of the psyllids to uptake DNA was investigated by adding a 724 bp GFP PCR product to the artificial diet at a concentration of 50 ng/µL. The 724 bp PCR product corresponding to GFP sequence was amplified from plasmid pJL24 using the primers PacI-GFP left: 5′- cggttaattaaatggctagcaaaggagaagaac-3′ and JAL34 : 5′-tttgcggccgcttatttgtagagctcatccatg-3′. The presence of the 724 bp GFP DNA was determined by polymerase chain reaction with the same primers on DNA extracted from the psyllids after they were allowed to feed for 48h.

For the mortality bioassays, dsRNA concentrations were standardized and diluted in the artificial diet and used for feeding assays. Groups of 30 teneral adult psyllids were used for each treatment. Psyllid mortality was scored daily for 4 days. For the mRNA knockdown experiments, the dsRNAs were added to the artificial diet at a concentration of 700 ng/µL and siRNAs were added at a concentration of 100 ng/µL. After the teneral adult psyllids were allowed to feed for 72 h, total RNAs were isolated from individual live psyllids using TRIzol (Invitrogen, catalog NO. 15596-018) and used for cDNA preparation and quantitative real-time PCR. In some cases using *BC-Actin* and GFP dsRNAs for feeding, total RNAs were isolated from the guts of a pool of ∼30 psyllids.

### 6. Quantitative real time PCR (qRT-PCR)

The effect of dsRNAs or siRNAs on target mRNA levels was quantified by quantitative real-time PCR using Fast SYBR Green Master Mix (Applied Biosystems). In order to avoid RT-PCR artifacts resulting from the input dsRNAs, primers for qRT-PCR were designed to detect target mRNAs by amplifying sequences that lay outside of the input dsRNA sequences. *B. cockerelli* rRNA (accession number GQ249868) was used as an internal control for normalization after validation. GFP dsRNA or GFP siRNAs was used as a control treatment. The relative gene expression data were analyzed using the relative 2^-ΔΔCT^ method [26]. Briefly, qPCR experiments were performed for the target transcript and internal control rRNA in replicate using the primers described in Table S1. The reaction mixture contained 7.5 µL SYBR Green Master Mix, 0.5 µL 10 µM forward primer, 0.5 µL 10 µM reverse primer and 3 µL cDNA template in a final reaction volume of 18 µL. 40 cycles of reaction is performed for 3 s at 95°C and 30 s at 60°C. The ΔCT value for individual samples was obtained by subtracting average Ct (cycle threshold) value for rRNA from average Ct value for the target transcript. One of the samples in control groups, GFP dsRNA or siRNA-fed psyllids, was selected as a reference sample. The ΔΔCT value for individual samples was obtained by subtracting ΔCT value for the reference sample from the ΔCT value of the test sample. The relative abundance of target transcript in comparison to rRNA level in each sample is represented by the 2^-ΔΔCT^ value. The average value and standard error value (SE value) of the 2^-ΔΔCT^ value in treatment and control groups were calculated separately, and the statistical analyses were performed between these two groups.

### 7. Statistical analysis

Experimental data from dsRNA feeding assays and qPCR results were analyzed by multiple tests including the Least Significant Difference (LSD) test, Ryan-Einot-Gabriel-Welsch (REGW) Multiple Range Test, Tukey's Studentized Range (HSD) Test, and the Bonferroni (Dunn) t Tests using SAS 9.1 software. We used the results from Bonferroni (Dunn) t Tests as it yielded the most conservative results and the statistical differences are shown as different letters or asterisks (*).

### 8. Small RNA analysis

RNA hybridization analysis was used to determine if sequence-specific siRNAs were present in psyllids following dsRNA acquisition. Small RNAs were extracted from psyllids after a 72 h feeding period, by using the mirVana PARIS small RNA isolation kit (Ambion, catalog NO. AM1556) according to the manufacturer's protocol. As a positive control, the *Tobacco mosaic virus* (*TMV*) vector pJL36[27], containing *BC-Actin, BC-ATPase* or *GFP* sequence was agro-infiltrated into *N. benthamiana* plants and small RNAs were isolated 2 weeks post-infiltration. Small RNA hybridization was performed according to the method described previously [28]. Briefly, 1 µg small RNAs were separated on denaturing 8M urea 15% polyacrylamide gels and transferred to nylon membranes (Amersham Hybond™-NX). The pGEM®-T Easy plasmids containing *GFP*, *BC-Actin* or *BC-ATPase* sequences were digested with restriction endonucleasesAvrII, PstI or NcoI, respectively, and the linearized plasmids were used as templates for the labeling reaction described below. ^32^P-UTP-labeled negative strand *GFP*, *BC-Actin* or *BC-ATPase* RNA transcripts were generated *in vitro* using SP6, T7 or SP6 RNA Polymerase (Ambion, catalog NO. AM1320), respectively. Transcripts were fragmented to ∼300nt in 120mM Na_2_CO_3_ : 80mM NaHCO_3_ solution at 65°C for 30 min and used as probes for hybridization. Hybridization was performed in ULTRAhyb®-Oligo hybridization buffer (Ambion; catalog NO. AM8663) at 42°C overnight and the membrane was washed twice in NorthernMax® Low Stringency Wash Buffer (Ambion, catalog NO. AM8673) at 42°C for 15 min. Blots were removed, drained, wrapped in SaranWrap and exposed to X-ray film at −80°C. The exposure times for *BC-Actin*, *BC-ATPase* and GFP were 15 h, 19 h and 8 h, respectively. MicroRNA Marker (New England Biolabs; catalog NO. N2102S) was used on the same gel to estimate RNA sizes.

## Results

### 1. Identification and tissue-specific transcription profile of partial gene sequences of *B. cockerelli*

When this study was initiated, only 40 nucleotide sequences were available for *B. cockerelli*. In contrast there were 19,605 EST sequences available for *D. citri,* including 6,201 sequences from midgut ESTs in the National Center for Biotechnology Information (http://www.ncbi.nlm.nih.gov). We took advantage of these *D. citri* EST sequences in order to amplify homologous sequences from *B. cockerelli*. To do this, the *D. citri* ESTs were aligned and analyzed by using the ArthropodEST EST analyses pipeline (http://bioinformatics.ksu.edu/ArthropodEST/) resulting in the retrieval of 1,904 contigs. 179 contigs from the midgut EST library were annotated and homologous to known protein sequences, among them 99 contigs were ribosomal or mitochondrial cDNAs and the other 80 sequences were further selected as targets of interest. We focused on the sequences that are highly expressed or specifically expressed in the gut, as they are likely to be good targets for orally-acquired anti-psyllid interfering RNAs because the midgut is one of the first barriers that the ingested RNAs will encounter following feeding, and some reports for other insects show that midgut-expressed mRNAs are susceptible to the effects of orally-delivered dsRNAs [10], [18], [23], [29].

The 80 *D. citri* contig sequences of interest were compared by nucleotide BLAST using the National Center for Biotechnology Information (http://www.ncbi.nlm.nih.gov) database, and consensus regions for homologous sequences were selected for designing primers (Table S1) for use in cloning the sequences from *B. cockerelli*. Twenty eight primers were successful yielding amplicons ranging from 150 bp to 700 bp. These cDNAs were cloned into pGEM®-T Easy vector (Promega) and sequenced (UC Davis sequencing facility). The resulting sequences showed 70–90% homology to their *D. citri* homologs, supporting our hypothesis that these two psyllid species are closely related (data not shown). We also verified expression for 23 of these sequences in *B. cockerelli* gut tissues by conventional RT-PCR (data not shown). Based on our preliminary results, putative homologues of *Actin*, *ATPase*, *Hsp70* and *CLIC* (Chloride Intracellular Channel) were further analyzed. We named these sequences as *BC-Actin*, *BC*-*ATPase*, *BC*-*Hsp70* and *BC*-*CLIC,* respectively. The sequences of *BC-Actin*, *BC*-*ATPase*, *BC*-*Hsp70* and *BC*-*CLIC* showed 91.7%, 79.8%, 83.4% and 81.5% sequence identity with their corresponding homologs in *D. citri* (Fig. S1).

**Figure 1:**
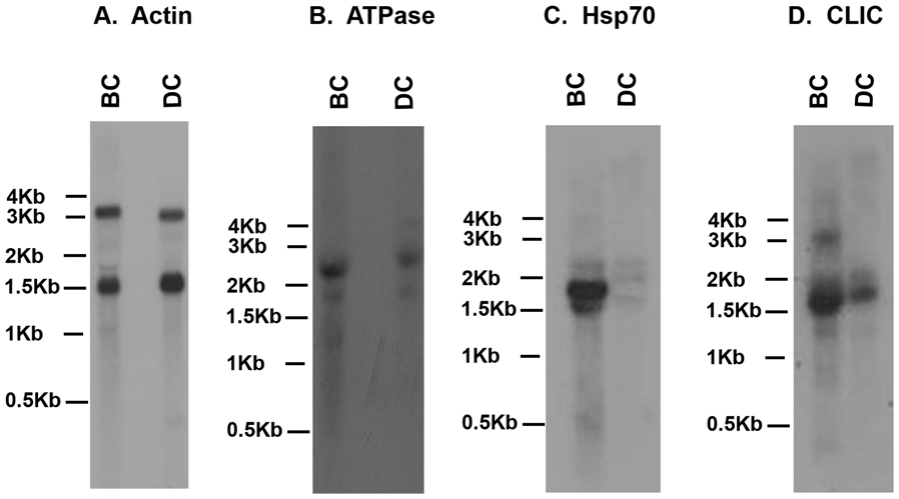
Northern blot analyses of *Actin, ATPase, Hsp70 and CLIC* mRNAs in *B. cockerelli* and *D. citri*. Total RNAs (1.5 µg) from *B. cockerelli (BC)* and *D. citri (DC)* were separated by denaturing agarose gel electrophoresis, transferred to nylon membranes and hybridized with ^32^P-UTP-labeled probes prepared using corresponding *B. cockerelli* sequences. 0.5–10 Kb RNA ladder (Invitrogen) was used on the same gel and the sizes are indicated. Labels A – D above each panel indicate the specific probe used for the corresponding blot. The exposure times for *BC-Actin*, *BC-ATPase*, *BC-Hsp70* and *BC-CLIC* were 15 h, 2 h, 38 h and 38 h, respectively.

**Figure 2:**
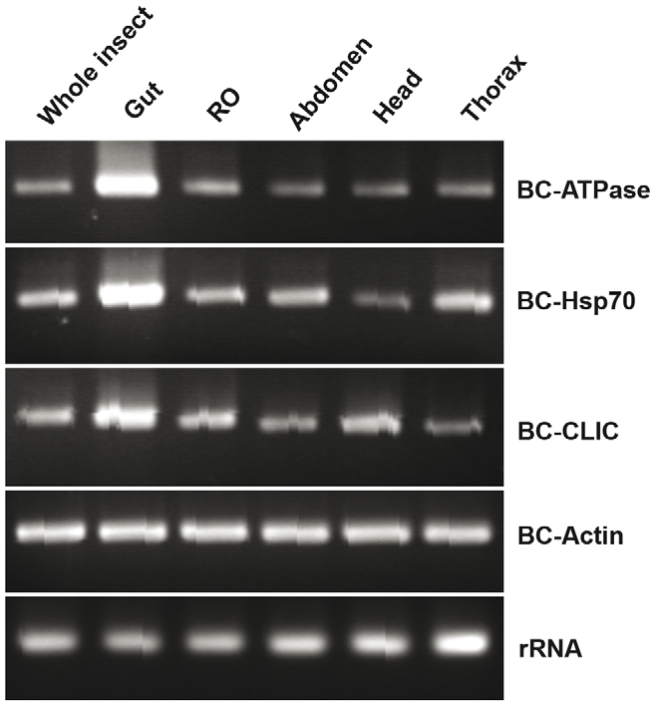
The relative accumulation of *B. cockerelli* mRNAs in different tissues. Total RNAs were extracted from dissected *B. cockereli* tissues including gut, head, abdomen, thorax, and reproductive organ (RO). cDNA was synthesized and semi-quantitative RT-PCR analyses were performed on *BC-Actin*, *BC-ATPase*, *BC-Hsp70* and *BC-CLIC*. Expression profile of *rRNA* was used as control. RT-PCR was carried out 30 cycles for *BC-ATPase*, *BC-Hsp70* and *BC-CLIC*, and 22 cycles for *BC-Actin* and *rRNA* using GoTaq polymerase. The PCR reactions are: initial denaturation at 94°C for 5 min, followed by cycles with 94°C (30 s), 55°C (30 s), 72°C (1 min), and a final extension for 7 min at 72°C.

To verify the presence of mRNAs for the selected sequences above, Northern hybridization analysis was performed using *B. cockerelli* sequence-specific probes and total RNAs from both *B. cockerelli* and *D. citri*. All probes showed positive hybridization signals for the *B. cockerelli* RNAs (Fig. 1). Interestingly, three of the four probes also gave hybridization signals for *D. citri* RNAs, and these were very similar in size to those for *B. cockerelli* (Fig. 1, panels A, B and D). Hybridization analysis for *BC-Actin* mRNAs gave a ∼1.5 kb RNA for both *B. cockerelli* and *D. citri*, and a larger fragment of ∼3 kb (Fig. 1A). In the pea aphid (*Acyrthosiphon pisum*), another phloem-feeding hemipteran insect, the reported actin (GenBank Accession NO. FP925933) mRNA size is 1554 bp, very close to the size of the most abundant RNA detected by us, further suggesting that this in fact represents the actin mRNA. The *BC-ATPase* probe recognized a RNA of 2–3 kb in *B. cockerelli* and a slightly larger RNA in *D. citri* (Fig. 1B). For *BC*-*Hsp70*, an 1.5–2 kb RNA was detected in *B. cockerelli*, but in *D. citri* the hybridization signal was very faint (Fig. 1C). The *BC*-*CLIC* probe hybridized with RNAs from both *B. cockerelli* and *D. citri,* but the signal for the latter was less intense (Fig. 1D). The above results clearly indicate the presence of mRNAs of selected sequences in *B. cockerelli*, and homology to three of the four corresponding *D. citri* mRNA. We next used semi-quantitative RT-PCR to approximate target mRNA abundance in psyllid tissues. rRNA level was used as internal control. *BC-Actin* mRNA was abundant in guts and all other tissues tested while *BC-ATPase*, *BC-Hsp70* and *BC-CLIC* mRNAs were relatively more abundant in psyllid guts than in other tissues including reproductive organs, abdomen, head and thorax (Fig. 2).

### 2. Injection of *BC-Actin* dsRNA caused mortality in *B. cockerelli*

Direct injection of dsRNAs has proven to be an effective way to demonstrate RNAi effects in numerous insect species [13]–[16]. We tested *B. cockerelli* nymphs and adults, and found the best survival (∼ 50% 1 day post injection) and ease of injection when using teneral adults and injecting 200 nL dsRNAs between the 2^nd^ and 3^rd^ abdominal segments. In two experiments comparing the survival of teneral adult psyllids that were injected with 200 nL of *BC-Actin* and control GFP dsRNAs, psyllids injected with *BC-Actin* dsRNA had higher mortality compared with psyllids injected with GFP dsRNA, beginning at two days post injection, and the mortality increased until the end of the experiments. When experiments were concluded six days post injection, psyllids injected with *BC-Actin* dsRNA showed 8% and 18% survival, whereas the survival was 42% and 57% for those psyllids injected with GFP dsRNA (Table 1).

**Table 1:** Mortality induced by injection of *BC-Actin* and GFP dsRNAs.

Injected dsRNA^1^	Total NO. of injected psyllids on day 0	No. and percentage of surviving individuals^3^					
		Day 1^2^	Day 2	Day 3	Day 4	Day 5	Day 6
GFP	40	21 (100%)	19 (90%)	16 (76%)	16 (76%)	13 (62%)	12 (57%)
*BC*-*Actin*	40	24 (100%)	19 (79%)	10 (42%)	9 (38%)	2 (8%)	2 (8%)
GFP	40	26(100%)	26 (100%)	23 (88%)	22 (85%)	21 (81%)	11 (42%)
*BC*-*Actin*	40	22 (100%)	19 (86%)	10 (45%)	8 (36%)	4 (18%)	4 (18%)

1. Each individual was injected with 200 nL of 100 ng/µL dsRNA.

2. About half of the injected psyllids would die on day 1 after injection and this is most likely caused by the injection procedure.

3. The percentages of surviving individuals on day 2 - 6 were based on the NO. of surviving individuals on day 1 for each case.

### 3. Feeding assays for *B. cockerelli*

**Figure 3:**
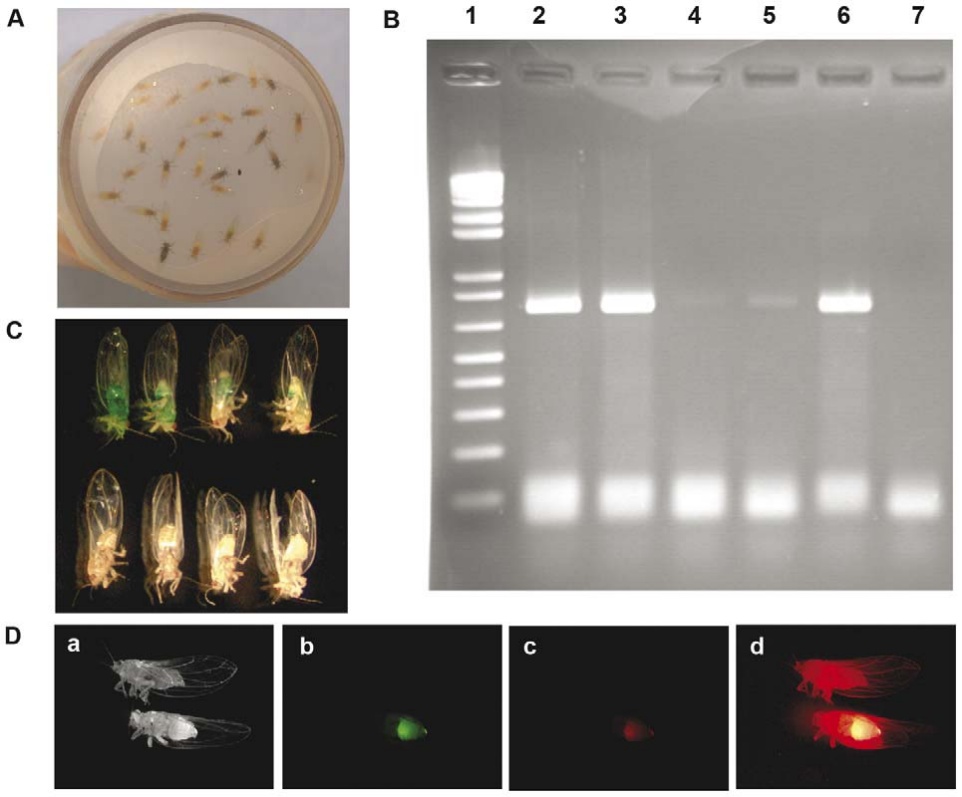
Validation of oral ingestion for *B. cockerelli*. (A) Psyllid feeding system. Teneral pale-colored adult psyllids are placed in plastic vials (25mm X 45mm). The feeding solution is 15% sucrose (w: v) sandwiched between two sheets of stretched parafilm. Plastic vials containing the psyllids were maintained at room temperature in the dark. (B) Psyllids were fed on 15% sucrose containing 50ng/µL of GFP PCR product. After 48h feeding, psyllids were collected, DNA extracted and analyzed by PCR. Lane 1, 1Kb plus DNA Marker; Lane 2 and 3, 0.004ng and 0.0004 ng GFP PCR product only as a positive control for PCR reaction. Lanes 4 and 5, PCR products from GFP DNA-fed psyllids; Lane 6, PCR product from sucrose-fed psyllid extract mixed with 0.004 ng GFP DNA; Lane 7, sucrose-fed psyllid used as negative control. (C) 1∶10 (v:v) dilution of green coloring dye was added to 15% sucrose and used for feeding. After 24h the psyllids were examined by low power light microscopy. Upper row is food coloring-fed psyllids and the lower row shows sucrose-fed psyllid controls. (D) 50 ng/µL Cy™3-labeled GFP dsRNA was supplied in 15% sucrose and fed for psyllids. After 48h feeding, the fluorescence was visualized by fluorescence microscopy (Leica DM5000B) equipped with a mercury lamp (ebq100, ISOLATED). In each panel, the lower psyllid was fed with Cy™3-labeled dsRNA and the upper psyllid was sucrose-fed psyllid control. Psyllids were visualized under white light (a) or under fluorescent light of different excitations at 460nm, 480nm and 436nm (b-d).

Delivery of dsRNAs by injection was tedious and caused high mortality. These experiments, however, demonstrated that RNAi effects could be induced in recipient psyllids. In order to utilize an easier and more practical dsRNA delivery approach, we developed an oral dsRNA delivery system for psyllids. The oral delivery system was based on a liquid artificial diet containing 15% (w:v) sucrose sandwiched between two layers of Parafilm. The Parafilm was stretched over a clear plastic vial (25 mm × 45 mm) in which the psyllids were housed and served as a feeding chamber. The psyllids readily congregated on the parafilm and imbibed on the artificial diet (Fig. 3A). In order to confirm that the psyllids were indeed feeding, three different markers were used. First, a 724 bp GFP PCR product was supplied in the artificial diet. After 48h feeding, DNA was purified from live psyllids and used as template for PCR detection of the GFP DNA. In Fig. 3B, lanes 2, 3 and 6 represent positive controls and lanes 4 and 5 are results for the GFP DNA-fed psyllids. Weak but clear bands are visible in lanes 4 and 5, but not in sucrose-fed psyllid controls (lane 7). Second, when the artificial diet contained green food coloring, green psyllids were easily seen after 24 h of feeding (Fig. 3C, top row). Third, fluorescent-labeled GFP dsRNAs were supplied in the sucrose solution. After 48h of feeding, the Cy™3 label (fluorescence) was easily observed in the dsRNA-fed psyllids (Fig. 3D, bottom), and the localization appeared to be restricted in the abdomen.

### 4. Sequence-specific and dose-dependent *B. cockerelli* mortality induced by dsRNA feeding

**Figure 4:**
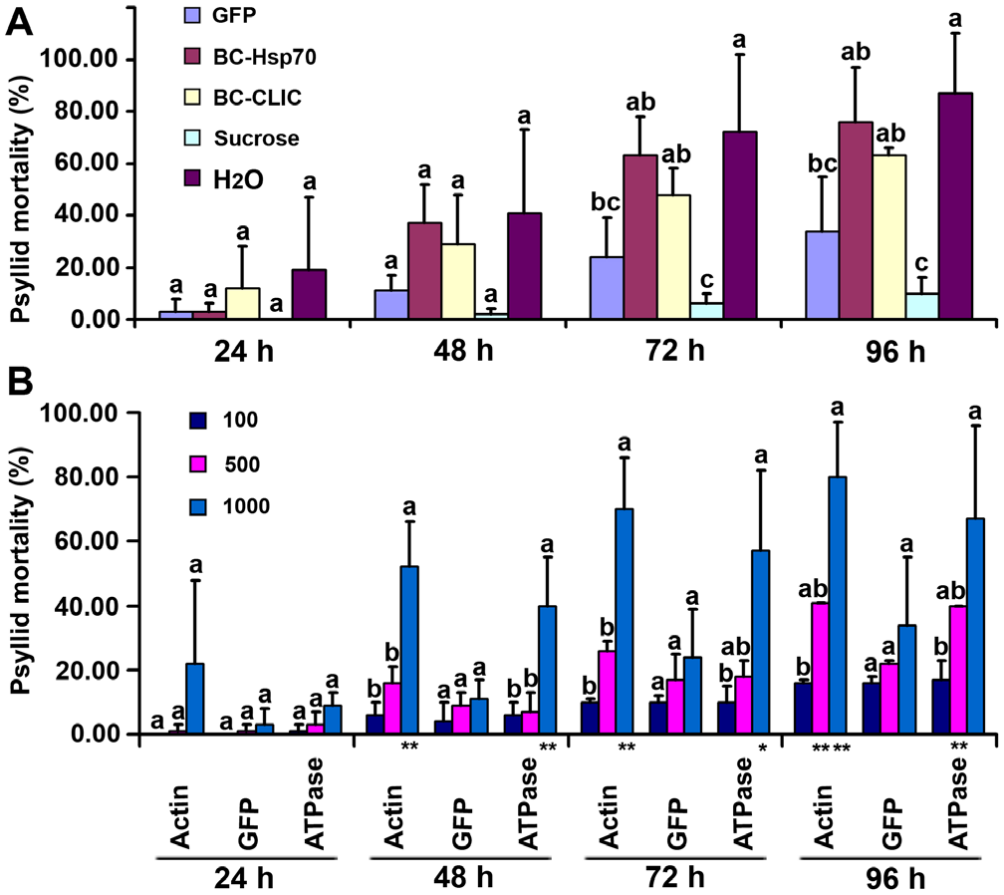
Dose - and time-response of psyllids allowed to feed on artificial diets containing dsRNAs. Groups of 30 teneral adult psyllids were used for each treatment. The experiments were repeated three times. 15% sucrose was used as a food control and H_2_O was used as a non-food control for psyllid mortality. GFP dsRNA was used as a dsRNA control. The Bonferroni (Dunn) t-test was used for statistical analysis. (A) show mortality by feeding 1000 ng/µL of *BC-Hsp70* and *BC-CLIC* dsRNA over time. Statistical differences between treatments at the same time points are shown by different letters (P<0.05). (B) Shows mortality induced by decreasing concentrations (1000ng/µL, 500 ng/µL, 100ng/µL) of *BC-Actin* or *BC-ATPase* dsRNAs. Statistical differences in mortality rates were evaluated between test dsRNAs and GFP dsRNA at the same concentrations and time points. Single asterisk indicates P<0.05, and double asterisks indicate P<0.01. Statistical differences are also shown by different letters between different concentrations for the same dsRNA at the same time points. In Figure (A) and (B) the same data are used for GFP 1000.

We used this feeding system to evaluate dsRNAs of different *B. cockerelli* sequences for their abilities to induce mortality in *B. cockerelli*. The dsRNAs were supplied at the concentration of 1000 ng/µL in the artificial diet and mortality was monitored daily for a 4 day feeding period. GFP dsRNA at the same concentration, and feeding solutions of 15% sucrose, or water only were used as controls for each experiment. Psyllids rapidly died when fed only on water, showing 35% mortality after 48 h and up to 97% mortality after 96 h, whereas less than 10% mortality was seen for the diet of only 15% sucrose after 96 h, demonstrating again the efficacy of our feeding system (Fig. 4A). Among the 10 dsRNAs evaluated here, six did not show differences compared to psyllids fed on the GFP dsRNA (Fig. S2, Table S2), whereas four dsRNAs gave higher mortality (Fig. 4). These were dsRNAs for *BC-Actin*, *BC-ATPase*, *BC-Hsp70* and *BC-CLIC*.

We also compared dsRNA concentration effects on psyllid mortality for *BC-Actin* and *BC-ATPase*. GFP dsRNAs at the same concentrations as the test dsRNAs were used as controls. Significantly more mortality was induced by the *BC-Actin* and *BC*-*ATPase* dsRNAs at 1000 ng/µL and 500 ng/µL compared with GFP dsRNA, but differences were not significant at the lowest concentration tested, 100 ng/µL (Fig. 4B). Furthermore, significantly greater and earlier mortality was seen at 1000 ng/µL than at 500ng/µL for *BC-Actin* and *BC-ATPase* dsRNAs, but not for GFP dsRNA (Fig. 4B). It is interesting to note that high concentrations (1000 ng/µL) of the GFP dsRNA control also caused higher mortality compared to the 15% sucrose control (Fig. 4A). Perhaps high concentrations of dsRNAs are able to initiate off-target, or RNAi pathway saturation effects deleterious to psyllids.

### 5. Oral feeding of dsRNAs and siRNAs decreased endogenous mRNA levels in *B. cockerelli*

**Figure 5:**
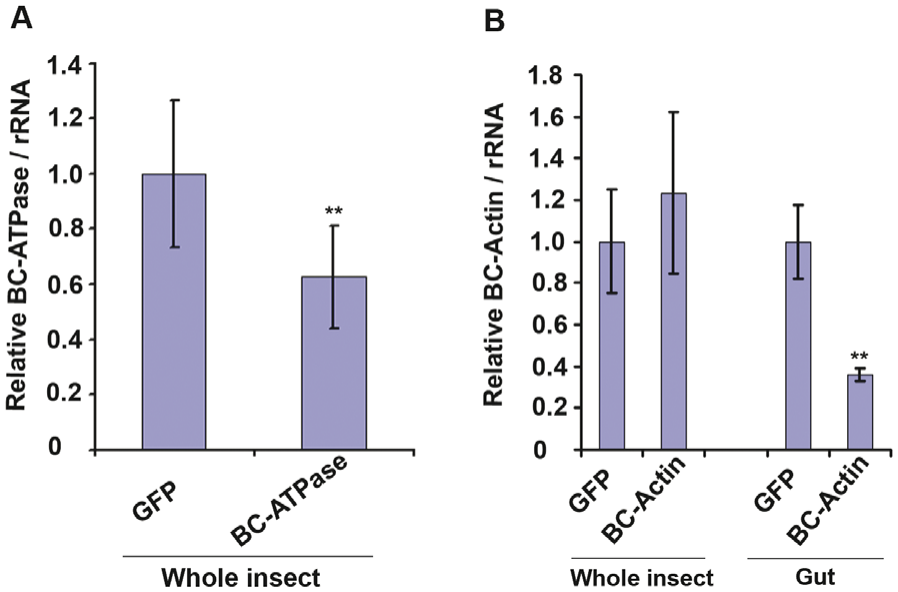
Knockdown of endogenous psyllid mRNAs by dsRNA feeding. The dsRNAs were added to the artificial diet at a concentration of 700 ng/µL. After the teneral adult psyllids were allowed to feed for 72 h, total RNAs were isolated from individual live psyllids by TRIzol method (Invitrogen) and used for cDNA preparation and quantitative real-time PCR. GFP dsRNA-fed served as control for each experiment. (A) show accumulation of *BC-ATPase* mRNAs in whole psyllid after feeding on dsRNAs for *BC-ATPase.* (B) show the mRNA abundance of *BC-Actin* mRNAs in psyllid fed on dsRNAs for *BC-Actin*. Whole psyllid and dissected guts from a pool of 30 psyllids were analyzed separately. The qRT- PCR results were normalized to the level of *B. cockerelli* rRNA. Mean values from experimental replicates were analyzed by using Bonferroni (Dunn) *t*-test to determine between-group significance. Double asterisks indicate differences at the P<0.01 level. The mRNA level in the GFP dsRNA-fed group was arbitarily designated as 1 in all experiments.

In order to determine if the orally-acquired dsRNAs were inducing RNAi effects, we used quantitative real-time PCR to assess target mRNA levels in psyllids. Psyllids were collected 72h after feeding on 700ng/µL dsRNAs and total RNAs were extracted from individual live psyllids. GFP dsRNA-fed psyllids were used as controls. Quantitative real time PCR results were normalized by quantifying the abundance of *B. cockerelli* rRNA (accession number GQ249868). These analyses revealed that the mRNA abundance of the *BC*-*ATPase* mRNA was decreased by 37%–65% after feeding on *BC*-*ATPase* dsRNA (Fig. 5A, Table 2). *BC*-*Hsp70* and *BC*-*CLIC* mRNAs also showed significant decreases in abundance when whole psyllids were analyzed (Table 2). These results demonstrated that mRNA knockdown is achievable for gut-highly-expressed mRNAs, and can be detected even when analyzing total psyllid RNAs. By contrast, analysis of total psyllid RNAs from the psyllids that fed on *BC-Actin* dsRNA did not show a reduction for the *BC-Actin* mRNA (Fig. 5B, Table 2). However, because we had observed ubiquitous expression of *BC-Actin* mRNA in all tissues we tested (Fig. 2), we asked if the *BC-Actin* mRNA knockdown in the guts could be masked by the total *BC-Actin* mRNA expressed in the whole body. Thus, we next compared *BC-Actin* mRNA abundance in the guts dissected from the psyllids that were fed on *BC-Actin* and control GFP dsRNAs. These analyses showed that *BC-Actin* mRNA was decreased by ∼70% in gut tissues of *BC-Actin* dsRNA-fed psyllids (Fig. 5B, Table 2). These results, together with the results from Fig. 3D, suggest that the psyllid gut is accessible to the ingested dsRNA, and therefore gut-expressed or gut-specific mRNAs could be more susceptible RNAi targets.

**Table 2:** Quantitative real-time PCR detection for endogenous *B. cockerelli* mRNAs after feeding of dsRNAs or siRNAs.

RNA type^1^	RNA sample from^2^	NO. of sample^3^	Sequence name^4^	Means ± SE in GFP sample^5^	Means ± SE in Test sample^5^	P Value^6^
dsRNA	WS	4	BC-Actin	1±0.25	1.21±0.30	0.4115
dsRNA	WS	6	BC-Actin	1±0.42	0.88±0.31	0.5776
dsRNA	Gut	2^+^	BC-Actin	1±0.04	0.29±0.02	0.0021**
dsRNA	Gut	2^+^	BC-Actin	1±0.18	0.36±0.03	0.0095**
dsRNA	WS	7	BC-ATPase	1±0.30	0.35±0.15	0.0002**
dsRNA	WS	5	BC-ATPase	1±0.27	0.63±0.18	0.0343*
dsRNA	WS	7	BC-ATPase	1±0.16	0.51±0.14	0.0002**
dsRNA	WS	4	BC-ATPase	1±0.25	0.42±0.39	0.0450*
siRNA	WS	8	BC-ATPase	1±0.27	0.56±0.20	0.0025**
siRNA	WS	6	BC-ATPase	1±0.37	0.39±0.09	0.0029**
dsRNA	WS	7	BC-Hsp70	1±0.25	0.34±0.14	0.0001**
dsRNA	WS	5	BC-Hsp70	1±0.26	0.63±0.12	0.0213*
dsRNA	WS	5	BC-CLIC	1±0.18	0.49±0.23	0.0049**
dsRNA	WS	5	BC-CLIC	1±0.07	0.82±0.06	0.0027**

1. Psyllids were fed with dsRNA or siRNAs as indicated.

2. Total RNA was isolated from individual psyllid or gut samples from a pool of 30 insects. WS, whole insect.

3. The number of psyllids used for qRT-PCR analysis in one experiment. For each experiment, the same numbers of GFP samples were used as controls for test sample. ^+^For gut sample, a pool of 30 psyllids was used for every treatment.

4. The name of sequences used for dsRNA feeding and mRNA detection by qRT-PCR.

5. The mRNA abundance of specific genes after introduction of corresponding dsRNA or SiRNA was shown as test sample, and the average value of the control GFP group was designated as 1. Expression of each mRNA was normalized to the level of rRNA in the same sample.

6. Difference between GFP group and test group was calculated and shown as P value using the Bonferroni (Dunn) t-test. Single asterisk indicates p<0.05 and double asterisk indicates p<0.01.

**Figure 6:**
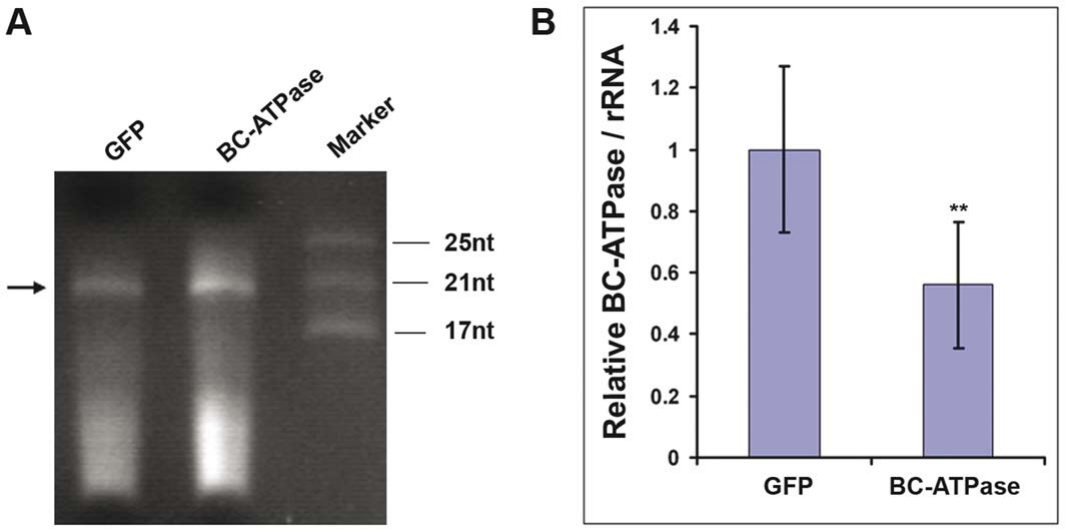
Knockdown of endogenous psyllid *BC-ATPase* mRNAs by siRNA feeding. (A) Small RNAs (21nt) were produced in vitro from GFP and *BC-ATPase* dsRNAs by digestion with ShorCut RNase III. The small RNAs were separated by 15% acrylamide gel electrophoresis and stained with ethidium bromide. The arrowhead indicates the position of 21nt siRNAs. The sizes of markers are indicated to the right. (B) show qRT-PCR results for *BC-ATPase* mRNAs after feeding on 100 ng/µL *BC-ATPase* siRNA for 72 h. GFP siRNA-fed psyllids served as control for treatment. The qRT-PCR results were normalized to the level of *B. cockerelli* rRNA. Differences between control GFP siRNA-treated samples and *BC-ATPase* siRNA-treated groups were calculated and shown as P values using the Bonferroni (Dunn) t-test. Double asterisks indicate p<0.01. The mRNA level in the dsGFP group was arbitarily designated as 1 in the experiment.

In plants, dsRNAs are processed into siRNAs by the RNAi machinery [30]. Since our long term goals include evaluating psyllid-targeted RNAi *in planta*, we next tested whether we could induce RNAi effects by delivering siRNAs. ∼21nt siRNAs for *BC*-*ATPase* and GFP sequences were generated *in vitro* (Fig. 6A), and added to the sucrose diet at final concentrations of 100ng/µL siRNA. The psyllids were collected at 72h after feeding and tested as above. Similar to our results for dsRNAs, *BC-ATPase* siRNAs also induced significant reductions in the target mRNA levels (Fig. 6B, Table 2).

### 6. Sequence specific siRNA was detected in the psyllids after oral acquisition of *BC-Actin* dsRNA

**Figure 7:**
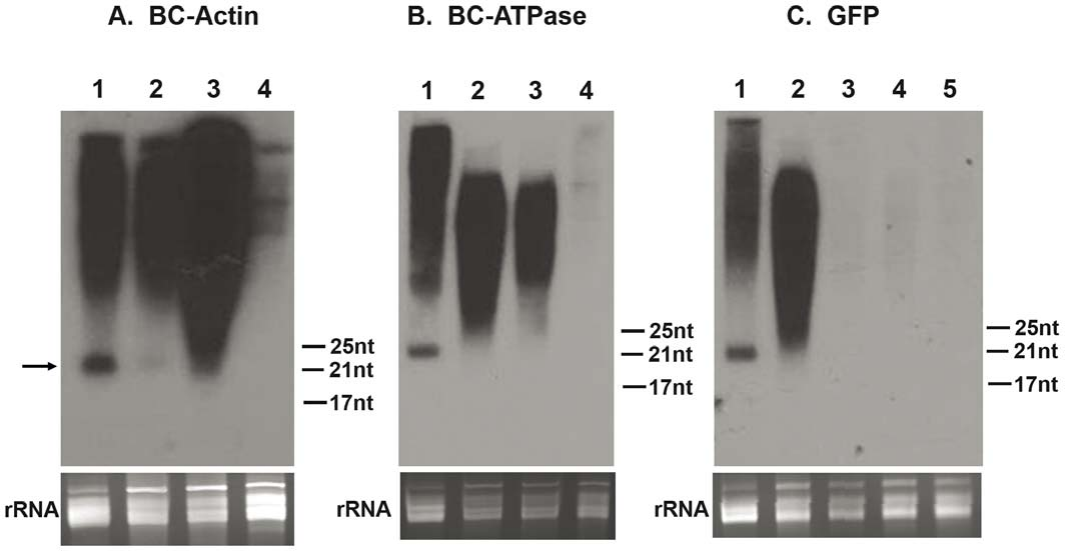
Northern blot detection of small RNA in *BC-Actin* dsRNA-fed psyllids. Teneral adult psyllids were allowed to feed for 72h on artificial diet containing 700 ng/µL of *BC-Actin*, *BC-ATPase* or GFP dsRNAs. Subsequently, small RNAs were isolated from group of ∼80 individuals, and 1 µg small RNA was separated on a 15% PAGE gel containing 8M Urea and transferred to a nylon membrane. ^32^P-UTP-labeled negative strand *BC-Actin* (A), *BC-ATPase* (B) or GFP (C) RNA transcripts were used as a probe for the corresponding blot. MicroRNA markers were analyzed on the same gel and sizes are indicated to the right of each blot. As a positive control, a *TMV* vector pJL36 containing *BC-Actin, BC-ATPase* or GFP fragment was agro-infiltrated into *N. benthamiana* and siRNAs were isolated after 2 weeks. (A) Lane 1, pJL36-*BC-Actin*-infected *N. benthamiana*; Lane 2, *BC-Actin* dsRNA-fed psyllids; Lane 3, GFP dsRNA-fed psyllids; Lane 4, sucrose-fed psyllids. The arrow on the left indicates the position of the siRNAs. (B) Lane 1, pJL36-*BC-ATPase*-infected *N. benthamiana*; Lane 2, *BC-ATPase* dsRNA-fed psyllids; Lane 3, GFP dsRNA-fed psyllids; Lane 4, sucrose-fed psyllids. (C) Lane 1, pJL36-GFP-infected *N. benthamiana*; Lane 2, GFP dsRNA-fed psyllids; Lane 3, GFP dsRNA-fed psyllids were transferred to and fed on tomato seedling for 48h; Lane 4, GFP dsRNA-fed psyllids were transferred to and fed on tomato seedling for 96h; Lane 5, sucrose-fed psyllids. The panels at the bottom show ethidium bromide stained rRNA as a loading control. The exposure times for *BC-Actin*, *BC-ATPase* and GFP were 15 h, 19 h and 8 h, respectively.

An important hallmark of RNAi activity is the generation of target-specific ∼21nt siRNAs by the RNA silencing machinery within the affected organism[6]. Therefore, we used RNA hybridization to investigate whether target-specific siRNAs were induced in dsRNA-fed psyllids. SiRNA accumulation was analyzed in *B. cockerelli* fed with sucrose solution containing *BC-Actin*, *BC-ATPase*, and GFP dsRNAs at a final concentration of 700 ng/µL. As plants respond systemically to virus infections by producing abundant siRNAs [31], as positive controls, we analyzed *N. benthamiana* plants infected with *Tobacco mosaic virus* (pJL36) containing the *BC-Actin, BC-ATPase* and GFP sequences. When sequence-specific probes were used, ∼21nt siRNAs were detected from these virus-infected plants (Fig. 7A, B, C, lanes 1). When dsRNA-fed psyllids were similarly analyzed, resolution of specific siRNAs was not as straightforward as for virus-infected plants. Hybridization signals appeared as a smear for the dsRNA-fed psyllids (Fig. 7A, B, lanes 2 and 3; 7C, lane 2), but not for the psyllids that fed only on 15% sucrose (Fig. 7A, B, lanes 4; 7C, lane 5). The smears in GFP dsRNA-fed psyllids were eliminated when the psyllids were transferred to tomato seedlings for 2 or more days immediately after feeding on dsRNA solutions (Fig. 7C, Lane 3 and 4), demonstrating that the smears represent sequence non-specific hybridization with degraded products of ingested dsRNAs, which are thus excreted after feeding on the seedlings. Interestingly, our RNA hybiridization analyses using the probes for *BC-Actin* did show weak but distinct siRNAs of ∼21nt in *BC-Actin* dsRNA-fed psyllids (Fig. 7A, lane 2). RNA samples from *N. benthamiana* plants infected with pJL36-GFP did not show small RNAs in the blot using *BC-Actin* probe, further confirming the sequence specificity of the probes for small RNA targets (Fig. S3). In contrast to *BC-Actin* dsRNA-fed psyllids, specific siRNAs were not detected in the psyllids fed on *BC-ATPase* and GFP dsRNAs (Fig. 7B, lane 2; 7C, lane 2) when the probes for *BC-ATPase* and GFP, respectively, were used. We also used qRT-PCR to show that the *BC-ATPase* mRNA is ∼10 times less abundant than *BC-Actin* mRNA even in the gut tissues (Fig. S4). These data ssuggest that the accumulation or stability of siRNAs in dsRNA-fed psyllids may be influenced by the abundance of endogenous mRNA.

## Discussion

In this study, we demonstrated the induction of specific RNAi effects in the potato/tomato psyllid (*B. cockerelli*) by injection and oral acquisition of dsRNA and siRNA inducers. Injection of teneral adult *B. cockerelli* with dsRNA corresponding to *BC-Actin*, led to increased mortality relative to control psyllids injected with GFP dsRNA. Injection of dsRNA corresponding to *BC-ATPase* also resulted in ∼ 30% reduction of the endogenous BC*-ATPase* mRNA level, verifying target specificity (Table S3). The injection procedure is very laborious and resulted in high mortality of recipient psyllids (∼50%). Injection is a powerful but inefficient means to screen large numbers of target sequences for RNAi activity, especially for a small and delicate insect such as the potato/tomato psyllid, but it is commonly used for fundamental studies of RNAi in insects and we included it here as a standard approach.

To overcome the limitations of injection, an artificial feeding system was developed for *B. cockerelli.* The use of food coloring and Cy™3-labeled dsRNA allowed us to confirm that psyllids actively fed on test solutions. We also found that dsRNAs were stable in the 15% sucrose artificial diet (data not shown), as is consistent with other studies [32]. Using this feeding system, we were able to direct RNAi activity against mRNAs for the homologues of *B. cockerelli Actin, ATPase, Hsp70*, and *CLIC*. In our feeding experiments, we could only detect significant RNAi effects at high dsRNA concentrations such as 500 ng/µL or 1000 ng/µL, as is consistent with the reports in other insects such as light brown apple moth (*Epiphyas postvittana*) and pea aphid (*Acyrthosiphon pisum*) [18], [33]. If high dsRNA concentrations were required for RNAi effects in target insects, this might compromise the practical application of RNAi approaches for insect control. However, significant knockdown of target mRNA was also detected by us after feeding *in vitro*-prepared siRNA at lower concentrations (100 ng/µL). It is also interesting to note that a statistically significant down-regulation for the *BC-Actin* mRNA, a globally-expressed housekeeping gene, was only detected in psyllid gut tissues but not in whole psyllids. This may suggest that even though robust RNAi activity was induced in guts (presumably in cells where dsRNAs were absorbed), the RNAi effects were not detected in distal tissues, possibly due to the failure of systemic RNAi responses in *B. cockerelli*. In contrast, our data showed that *BC-ATPase*, *BC-Hsp70* and *BC-CLIC* are predominantly expressed in psyllid guts, and RNAi effects against their mRNAs were apparent even when total RNAs from whole psyllids were analyzed. To confirm that the mRNA reductions seen in above experiments were due to RNAi, we were able to identify the presence of gene-specific small-interfering RNAs (siRNAs) for the abundant mRNA for *BC-Actin*. This is consistent with our previous data showing that *actin*-specific ∼21nt siRNAs were induced by transfection of specific dsRNAs in cultured cells of *H. vitripennis*, another hemipteran insect [28]. For a less abundant mRNA like that for *BC-ATPase*, we did not detect specific siRNAs. This could be due to the fact that *BC-ATPase* mRNA is ∼10 times less abundant than is the *BC-Actin* mRNA, even in the gut tissues (Fig. S4), and the accumulation or the stability of the siRNAs derived from the dsRNA might be affected by the abundance of endogenous mRNA. It should also be noted that the accumulation level of the siRNAs seen here was very low, maybe owing to the observation that the RNA interference effects are restricted to the gut tissues. When the foreign sequence GFP dsRNAs were fed to psyllids, we did not see GFP-specific siRNAs, but a hybridization signal as a smear, likely indicating non-specific degradation of the acquired GFP dsRNA. The accumulation of siRNAs following the introduction of dsRNAs or from virus infection, has been shown in other insect orders including the Lepidopteran species *Helicoverpa armigera* [23], [24], *Spodoptera litura* [34] and *Bombyx mori* [35], and in the Dipteran species *Aedes aegypyti* [36]. Next generation sequencing efforts also confirmed the presence of specific 21nt siRNAs in cultured cells of different mosquito species including *Aedes aegypti* [36], *Culex quinquefasciatus* [37] and *Anopheles gambiae* [38] after virus infections. The hemipteran species *Acyrthosiphon pisum* possesses homologues of Dicer 1 and Argonaute 2b, both of which are components of the RNA silencing complex in *Drosophila*, suggesting the existence of the RNAi pathway in hemipteran species [39], and thus our finding of siRNA production and observed RNAi effects are to be expected.

The product of the *V-ATPase* gene transports protons across intracellular and plasma membranes and plays important cellular roles including the regulation of intracellular pH and maintenance of membrane potential in insects [40]. *V-ATPase* mRNAs have been shown to be susceptible to RNAi effects, even leading to mortality in insects including *Acyrthosiphon pisum* [21], *Diabrotica virgifera virgifera LeConte* [22], *Leptinotarsa decemlineata* [41] and *Bemisia tabaci* [42]. The target efficiency of the *ATPase* gene family for RNAi effects is further confirmed in the present study, as we show that both injection and oral delivery of *BC-ATPase* dsRNA significantly decreased the endogenous *BC-ATPase* mRNA level. SiRNAs are also reported to be effective inducers of RNAi activity in several insect species [43]–[45]. For example, in the whitefly, *Bemisia tabaci*, oral ingestion of *V-ATPase* siRNAs that were generated by RNase III digestions showed lethal effects [42]. In the present study, we show that oral introduction of *in vitro*-generated siRNAs suppressed the accumulation of endogenous *BC*-*ATPase* mRNA, indicating that knockdown of insect mRNAs can be induced by oral delivery of siRNAs, further supporting the existence of RNAi-mediated silencing pathways in psyllids. Most of the sap-sucking insects including *B. cockerelli* feed on the plant phloem sap as it is transported within sieve tubes. Small RNAs, including siRNAs and microRNAs, have been reported to be present in the plant phloem [46], [47]. Therefore, the efficacy and specificity of orally-delivered siRNAs for insect RNAi will be of a tremendous value for future applications of RNAi via transgenic plants, especially for the phloem feeders like *B. cockerelli*.

In conclusion, our results demonstrate the induction of RNAi effects in the potato/tomato psyllid (*B. cockerelli*). This is important and supports the possibility of potentially using RNAi-based strategies for controlling this psyllid, and perhaps even the most-serious disease of citrus, HLB, in which the pathogen (*Candidatus* Liberibacter asiaticus) is transmitted by the Asian citrus psyllid (*D. citri*).

## Supporting Information

Figure S1 **Alignment of the** ***B. cockerelli BC-Actin*** **,** ***BC-ATPase*** **,** ***BC-Hsp70*** **and** ***BC-CLIC*** **cDNA partial sequences with their homologues from** ***D. citri*** **.** “*” indicates nucleotides conserved in *B. cockerelli* and *D. citri*.
(PDF)

Figure S2 **Psyllid mortality over time after ingestion of dsRNAs.** 1000 ng/µL dsRNAs are supplied in artificial food (15% sucrose), and 30 teneral adult psyllids were used for each treatment. Mortality was scored daily for 4 days. The experiments were repeated three times.
(TIF)

Figure S3 **Sequence specificity of** ***BC-Actin*** **probe for small RNA hybridization.** TMV vector pJL36 containing *BC-Actin* or GFP sequences were agro-infiltrated into *N. benthamiana* plants and siRNAs were isolated after 2 weeks. 1 µg of the small RNA fraction was separated on a 15% PAGE gel containing 8M Urea and transferred to a nylon membrane. A ^32^P-UTP-labeled negative strand *BC-Actin* RNA transcript was used as a probe for the corresponding blot. MicroRNA markers were analyzed on the same gel and sizes are indicated to the right of the blot. (A) Lane 1, pJL36-BC-Actin-infected *N. benthamiana*; Lane 2, pJL36- GFP-infected *N. benthamiana*. Exposure time for the blot is 3.5 h. (B) Longer exposure (34 h) of lane 2 still did not detect siRNAs, demonstrating probe specificity.
(TIF)

Figure S4 **Analysis of** ***BC-Actin*** **and** ***BC-ATPase*** **mRNA levels in psyllid guts and whole insect.** Quantitative real-time PCR was performed using qRT-PCR primers for *BC-Actin*, *BC-ATPase* and *rRNA* using the cDNA of whole insects and dissected guts of *B. cockerelli*. pGEM®-T Easy plasmids containing fragments of *BC-Actin*, *BC-ATPase* and *rRNA* served as standards for copy number calculation. The plasmid concentration was converted into copy number and a dilution series of each plasmid with copy number from 10^7^ to10^2^ was used as the DNA standard for quantitative real-time PCR. Standard curves were drawn by plotting the threshold cycle (CT) against the natural log of the copy number of plasmid molecules. *BC-Actin* and *BC-ATPase* mRNA levels in each sample are indicated as copy number per 10,000 rRNA mRNA molecules.
(TIF)

Table S1 **PCR primers used for this study.**
(DOC)

Table S2 **Annotation for the sequences used for feeding experiments in Figure S2.**
(DOC)

Table S3 **Quantitative real-time PCR detection for endogenous** ***BC-ATPase*** **mRNA after injection of dsRNAs.**
(DOC)
