# Supplementary material for: Oral Delivery of Double-Stranded RNAs and siRNAs Induces RNAi Effects in the Potato/Tomato Psyllid, Bactericerca cockerelli
Source: PLoS One. 2011 Nov 16;6(11):e27736. doi: 10.1371/journal.pone.0027736 (PMC3218023; doi:10.1371/journal.pone.0027736)
Supplement: Figure S1 — Alignment of the B. cockerelli BC-Actin , BC-ATPase , BC-Hsp70 and BC-CLIC cDNA partial sequences with their homologues from D. citri . “*” indicates nucleotides conserved in B. cockerelli and D. citri. (PDF) [file pone.0027736.s001.pdf]

Alignment of DC-Actin with BC-Actin

```
DC-Actin        TATTGTGKGACGACGACGTAGCCGCTTTG-TCGTG-ACAATGGT-CCGGTATK-GCAAG-
BC-Actin        TAT-GTGTGACGACGACGTAGCCGCTTTGGTCGTGGACAATGGTTCCGGTATGTGCAAGG
                *** *** ********************* ***** ******** *******  ***** 

DC-Actin        CCGGATTCGCCGA-GATGACGCTCCCCGAGCTGTCTTCCCCTCAATCGTCGGTAGACCC-
BC-Actin        CCGGATTCGCCGGTGATGACGCCCCCAGAGCCGTCTTCCCCTCAATCGTCGGTAGACCCC
                ************  ******** *** **** *************************** 

DC-Actin        GTCATCAGGGTGTCATGGTGGGTATGGGTCAAAAAGACTCCTACGTCGGTGATGAGGCTC
BC-Actin        GTCATCAGGGTGTCATGGTGGGTATGGGTCAAAAAGACTCCTACGTCGGTGATGAGGCTC
                ************************************************************

DC-Actin        AATCCAAGAGAGGTATCCTCACC-TGAAATACCCCATTGAGCACGGTATCATCACCAACT
BC-Actin        AGTCCAAGAGAGGTATCCTCACCCTGAAATACCCCATCGAGCACGGTATCATCACCAACT
                * ********************* ************* **********************

DC-Actin        GGGACGATATGGAAAAGATCTGGCATCACACTTTCTACAATGAGCTCCGTGTTGCCCCTG
BC-Actin        GGGACGACATGGAGAAGATCTGGCATCACACCTTCTACAACGAGCTGAGAGTCGCCCCCG
                ******* ***** ***************** ******** *****  * ** ***** *

DC-Actin        AGGAGCACCCCATCCTGCTGACCGAAGCTCCCCTCAACCCCAAGGCCAACAGAGAAAAGA
BC-Actin        AGGAGCACCCCATCCTGCTGACGGAGGCACCCCTCAACCCCAAGGCCAACAGAGAGAAGA
                ********************** ** ** ************************** ****

DC-Actin        TGACCCAGATCATGTTTGAAACCTTCAACACCCCCGCCATGTATGTTGCCATCCAGGCTG
BC-Actin        TGACCCAGATCATGTTTGAGACGTTCAACACCCCCGCCATGTACGTCGCCATCCAGGCTG
                ******************* ** ******************** ** *************

DC-Actin        TGCTCTCCCTGTACGCTTCCGGTCGTACCACTGGTATCGTGCTTGACTCCGGAGATGGTG
BC-Actin        TGCTCTCCCTGTACGCCTCTGGTCGTACCACCGGTATCGTGCTCGACTCTGGAGATGGTG
                **************** ** *********** *********** ***** **********

DC-Actin        TCTCCCACACCGTCCCCATCTATGAAGGTTACGCCCTTCCCCACGCCATCCTCCGTCTGG
BC-Actin        TCTCCCACACCGTCCCCATCTATGAAGGTTACGCCCTCCCCCACGCCATCCTCCGTCTGG
                ************************************* **********************

DC-Actin        ATCTGGCTGGTCGTGACTTGACCGACTACCTGATGAAGATCCTCACCGAGAGAGGTTACT
BC-Actin        ATCTGGCTGGTCGTGACTTGACCGACTACCTGATGAAGATCCTCACCGAGAGAGGTTACT
                ************************************************************

DC-Actin        CTTTCACCACCACCGCTGAGCGTGAAATCGTTCGTGACATCAAGGAGAAGCTGTGCTACG
BC-Actin        CCTTCACCACCACCGCTGAGCGGGAAATCGTCCGTGACATCAAGGAGAAGCTCTGCTACG
                * ******************** ******** ******************** *******

DC-Actin        TCGCCCTGGACTTTGAACAGGAAATGGCCACCGCTGCTGCCTCCACTTCTCTGGAGAAAT
BC-Actin        TGGCCCTGGACTTCGAGCAGGAGATGGCCACCGCCGCCGCCTCCACCTCCCTGGAGAAGA
                * *********** ** ***** *********** ** ******** ** ********  

DC-Actin        CCTACGAACTTCCCGACGGTCAAGTCATCACCATCGGAAACGAAAGA-TTCCGTTGTCCC
BC-Actin        GCTACGAGCTGCCCGACGGACAAGTCATCACATCGGGAAACGAGAGAATTCCGTTGTCCC
                 ****** ** ******** ***********    ******** *** ************

DC-Actin        GAAGCTCTGTT-CCAACCTTCCTTCCTG
BC-Actin        GAGGCTCTGTTTCCAGCCTTCCTTCCTG
                ** ******** *** ************
```

Alignment of DC-ATPase with BC-ATPase

```
DC-ATPase        ACAGACGGAAGATATGCAACTGCATTGTACTCAGCAGCCACTAAACTGAAGCAGTTGGAT
BC-ATPas         ACTGAAGGAAGATATGCCACTGCATTATACTCTGCGGCAACAAAGCTGAAACAGTTGGAA
                 ** ** *********** ******** ***** ** ** ** ** ***** ********

DC-ATPase        GGTGTTGAAAAGGAATTGATTTCTTTCCAACAAGCCTTGAAAACTGACGTGAAGTTCCGT
BC-ATPas         GGTGTTGAAAAAGAGTTGATCTCCTTCCAGAAAGCCTTGAAAACTGATGTGAAATTCCGT
                 *********** ** ***** ** *****  **************** ***** ******

DC-ATPase        GACTTTGTGTTAGATCCCACCATCCAGAAGAGCCTCAAAATTGAAGCCCTCAAGATTGTG
BC-ATPas         GACTTTGTGCTGGACCCTACCATTAAGAAGACACTTAAAATCGAAGCCCTTAAGTCAATA
                 ********* * ** ** *****  ******  ** ***** ******** ***    *

DC-ATPase        GGTCAAAAGAAAAACTTCTCTGCTGCCTCCATCAATCTGTTGGCTTTGTTGGCAGAGAAT
BC-ATPas         GGTCAGAAGCAAAAGTTCTCTGCAGCCTCTATTAACTTGCTCTCACTGTTAGCTGAGAAT
                 ***** *** **** ******** ***** ** **  ** *  *  **** ** ******

DC-ATPase        GGCAAAATCAAGAACATTGATGGAGTGATCAATAACTTCTCAATCATTATGGCAGCTCAT
BC-ATPas         GGAAGGCTCAAGAGCATTGATGGAGTCATCAACAACTTTTCCATTATCATGGCAGCCCAC
                 ** *   ****** ************ ***** ***** ** ** ** ******** **

DC-ATPase        AGAGGAGACCTCCCTGTGGAAGTTATTACTGCTAGGCCTTTGGAAGAAGCTGACAAAAGT
BC-ATPas         AGAGGAGATCTGCCAGTGGAAGTTATCACTGCCAGGCCTCTGGATGATGCTGACAAGACA
                 ******** ** ** *********** ***** ****** **** ** ******** *

DC-ATPase        GAGCTGCAGTCCACCCTCAAATTGTTTGCCAAGAAAGGTGAAAATATCCTGTTGACCACC
BC-ATPas         GAGCTGCAATCCACCCTGAAACTTTTTGCCAAGAAGGGCGAGAACATTATTCTGACATCC
                 ******** ******** *** * *********** ** ** ** **  *  ****  **

DC-ATPase        AAAGTTGACCCTAGCATCATTGGAGGTATGATTGTCAGTGTGGGAGACAGGTACGTTGAC
BC-ATPas         AAAGTTGATCCCAGCATCATTGGAGGCATGATTGTCAGCATTGGTGACAAGTATGTCGAC
                 ******** ** ************** ***********  * ** **** *** ** ***

DC-ATPase        ATGAGTGTA
BC-ATPas         ATGAGTGTT
                 ********
```

```
Alignment of DC-Hsp70 with BC-Hsp70

DC-Hsp70        TTCGTGTGTGGGTGTGTTCCAACACGGTAAAGTGGAAATCATTGCCAACGACCAGGGTAA
BC-Hsp70        CTCCTGTGTGGGAGTTTTTCAACACGGAAAGGTAGAAATCATTGCAAATGACCAAGGAAA
                 ** ******** ** ** ******** ** ** *********** ** ***** ** **

DC-Hsp70        CAGAACCACACCCAGTTATGTTGCCTTCACCGACACAGAACGTTTAATCGGGGATGCTGC
BC-Hsp70        CAGGACCACACCCAGTTATGTTGCCTTCACTGACACTGAACGTCTAATAGGAGATGCTGC
                *** ************************** ***** ****** **** ** ********

DC-Hsp70        CAAAAACCAGGTGGCCATGAACCCCAACAACACCATCTTTGATGCTAAACGTTTAATCGG
BC-Hsp70        CAAAAACCAAGTAGCAATGAACCCCAACAACACCATTTTTGATGCCAAACGTTTAATAGG
                ********* ** ** ******************** ******** *********** **

DC-Hsp70        GCGCAAGTTTGATGATGCAACCGTACAGGCTGACATGAAGCACTGGCCATTCACTGTTGT
BC-Hsp70        CCGTAAGTTTGAAGATGCCACCGTTCAAGCTGACATGAAACATTGGCCCTTTGAAGTGAT
                 ** ******** ***** ***** ** *********** ** ***** **    **  *

DC-Hsp70        CAGTGATGGAGGCAAACCTAAAATCCAAGTTGAATACAAAGGAGAAACCAAGAGCTTCTT
BC-Hsp70        TAGTGATGGAGGCAAACCCAAGATACAAGTCAATTACAAAGGCGAGACCAAATCTTTCTT
                 ***************** ** ** *****  * ******** ** *****    *****

DC-Hsp70        CCCTGAAGAGGTATCCTCCATGGTGTTAACCA
BC-Hsp70        CCCAGAAGAAGTATCCTCCATGGTGTTGACCA
                *** ***** ***************** ****
```

Alignment of DC-CLIC with BC-CLIC

```
DC-CLIC         TGCTGAACTGAAAACGATTAGCCTCAAAGTGACCACAGTTGACATGCAGAAACCACCACC
BC-CLIC         TGCAGAGCTAAAAACCATTAGCTTGAAGGTTACAACAGTTGATATGCAGAAACCTCCACC
                *** ** ** ***** ****** * ** ** ** ******** *********** *****

DC-CLIC         AGATTTCAGAACAAATTTCGAGGCCACACCCCCTCCCATCCTGATAGACAATGGTCTGGC
BC-CLIC         TGATTTCAGAACAAACTTTGAAGCTACTCCACCACCAACTCTTATAGACAATGGCCTGGC
                 ************** ** ** ** ** ** ** ** *  ** *********** *****

DC-CLIC         AGTCCTAGAGAACGAGAAGATTGAACGTCACATCATGAAGAATGTACCGGGTGGTCACAA
BC-CLIC         TGTACTTGAAAACGAAAAAATTGAGCGTCATATCATGAAAAATGTACCTGGAGGACACAA
                 ** ** ** ***** ** ***** ***** ******** ******** ** ** *****

DC-CLIC         TCTGTTCGTACAAGACAAGGAAGTTGCCACACTCATTGAAAACCTGTATAGTAAACTGAA
BC-CLIC         CCTATTCGTGCAAGACAAGGAAGTTGCTGTTCTCATTGAGAACCTGTATAGTAAATTAAA
                 ** ***** *****************    ******** *************** * **

DC-CLIC         GCTGATGCTCCTGAAGAAGGACGATGTCAGCATAAACGCCCTCATGTCACATCTGCGCAA
BC-CLIC         ATTGATGCTCTTGAAGGAAGATGATGTCAGCATAAATGCCCTCATGTCACACCTTCGCAA
                  ******** ***** * ** ************** ************** ** *****

DC-CLIC         AATCAACGACCATCTCGGCAGAAAAGAGACCCGATTCCTAACCGGAGATACG
BC-CLIC         AATAAATGACCACCTTGGCAAGAAAGACACCCGCTTCCTAACTGGAGATACC
                *** ** ***** ** ****  ***** ***** ******** ********
```
