# Supplementary figures and images for: Oral Delivery of Double-Stranded RNAs and siRNAs Induces RNAi Effects in the Potato/Tomato Psyllid, Bactericerca cockerelli
Source: PLoS One. 2011 Nov 16;6(11):e27736. doi: 10.1371/journal.pone.0027736 (PMC3218023; doi:10.1371/journal.pone.0027736)

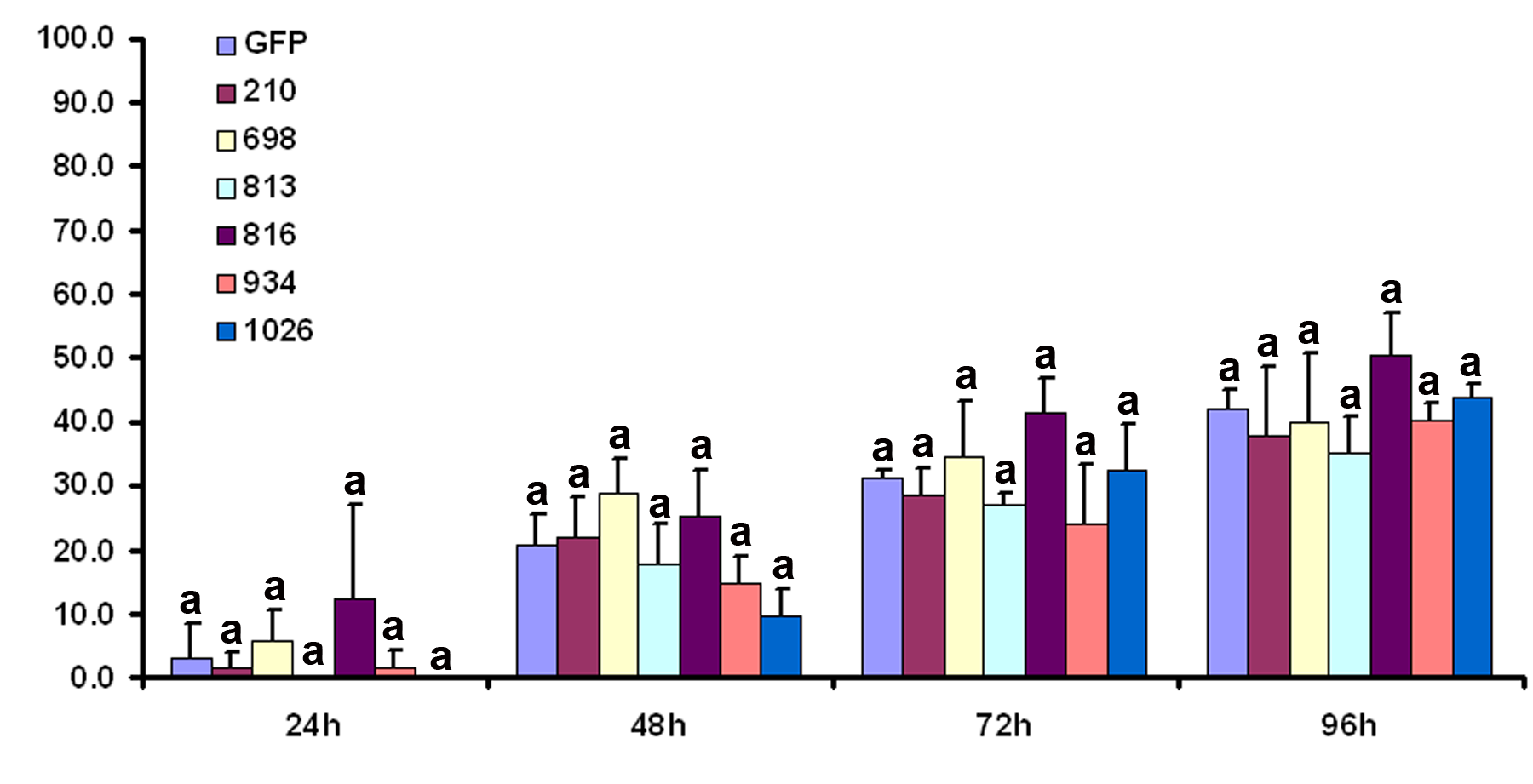

Supplement: Figure S2 — Psyllid mortality over time after ingestion of dsRNAs. 1000 ng/µL dsRNAs are supplied in artificial food (15% sucrose), and 30 teneral adult psyllids were used for each treatment. Mortality was scored daily for 4 days. The experiments were repeated three times. (TIF) [file pone.0027736.s002.tif]

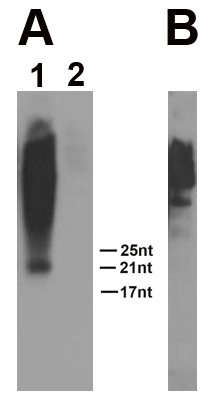

Supplement: Figure S3 — Sequence specificity of BC-Actin probe for small RNA hybridization. TMV vector pJL36 containing BC-Actin or GFP sequences were agro-infiltrated into N. benthamiana plants and siRNAs were isolated after 2 weeks. 1 µg of the small RNA fraction was separated on a 15% PAGE gel containing 8M Urea and transferred to a nylon membrane. A 32P-UTP-labeled negative strand BC-Actin RNA transcript was used as a probe for the corresponding blot. MicroRNA markers were analyzed on the same gel and sizes are indicated to the right of the blot. (A) Lane 1, pJL36-BC-Actin-infected N. benthamiana; Lane 2, pJL36- GFP-infected N. benthamiana. Exposure time for the blot is 3.5 h. (B) Longer exposure (34 h) of lane 2 still did not detect siRNAs, demonstrating probe specificity. (TIF) [file pone.0027736.s003.tif]

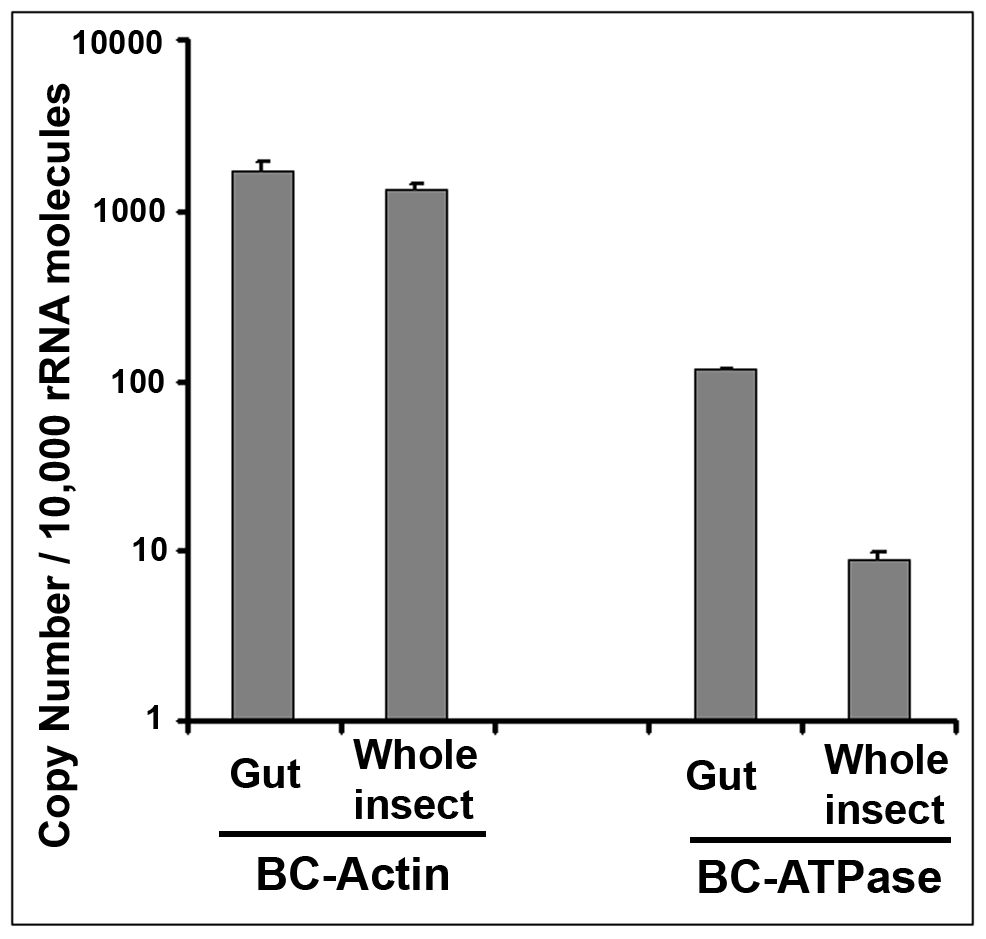

Supplement: Figure S4 — Analysis of BC-Actin and BC-ATPase mRNA levels in psyllid guts and whole insect. Quantitative real-time PCR was performed using qRT-PCR primers for BC-Actin, BC-ATPase and rRNA using the cDNA of whole insects and dissected guts of B. cockerelli. pGEM®-T Easy plasmids containing fragments of BC-Actin, BC-ATPase and rRNA served as standards for copy number calculation. The plasmid concentration was converted into copy number and a dilution series of each plasmid with copy number from 107 to102 was used as the DNA standard for quantitative real-time PCR. Standard curves were drawn by plotting the threshold cycle (CT) against the natural log of the copy number of plasmid molecules. BC-Actin and BC-ATPase mRNA levels in each sample are indicated as copy number per 10,000 rRNA mRNA molecules. (TIF) [file pone.0027736.s004.tif]
