# Supplementary material for: Oral Delivery of Double-Stranded RNAs and siRNAs Induces RNAi Effects in the Potato/Tomato Psyllid, Bactericerca cockerelli
Source: PLoS One. 2011 Nov 16;6(11):e27736. doi: 10.1371/journal.pone.0027736 (PMC3218023; doi:10.1371/journal.pone.0027736)
Supplement: Table S1 — PCR primers used for this study. (DOC) [file pone.0027736.s005.doc]

**Table** S1. PCR primers used for this study.

| **Sequence name^1^** | **Primer sequences^2^** | **Product size (bp)** |
| --- | --- | --- |
| **Primers for homologous sequences cloning for *B.cockerelli*** | | |
| *BC-Actin* | For: ATGTGTGACGACGACGTAGC | 807 |
|  | Rev: GGGGAGCGATGATCTTGATC |  |
| *BC-ATPase* | For: GTCAAGGCTCCAGTACAAGTATTTGGT | 489 |
|  | Rev: TCAGTGTATTTCTTGATTTTGCTG |  |
| *BC-Hsp70* | For: GGTATAGACTTGGGCACCACATA | 332 |
|  | Rev: AAGCTTCGGCAGTTTCCTTCATC |  |
| *BC-CLIC* | For: TATTTCATGGACCTGTACCTTC | 352 |
|  | Rev: TCGCAGTCAAAACAGCACAT |  |
| **Primers for dsRNA synthesis^3^** | | |
| *BC-Actin* | For: C*TAATACGACTCACTATAGG*GCGGCCGCCTCCCTGTACGCCTCTGGT | 364 |
|  | Rev: C*TAATACGACTCACTATAGG*GCGGCCGCGCAGCTCGTAGCTCTTCTCC |  |
| *BC-ATPase* | For: C*TAATACGACTCACTATAGG*GCGGCCGCTTGTGCTGGACCCTACCATT | 386 |
|  | Rev: C*TAATACGACTCACTATAGG*GCGGCCGCCAATCATGCCTCCAATGATG |  |
| *BC-Hsp70* | For: C*TAATACGACTCACTATAGG*GCGGCCGCGACCACACCCAGTTATGTTGC | 296 |
|  | Rev: C*TAATACGACTCACTATAGG*GCGGCCGCGGGAAGAAAGATTTGGTCTCG |  |
| *BC-CLIC* | For: C*TAATACGACTCACTATAGG*GCGGCCGCGCTAAAAACCATTAGCTTGAAGG | 344 |
|  | Rev: C*TAATACGACTCACTATAGG*GCGGCCGCAGGTGTGACATGAGGGCATT |  |
| *GFP* | For: C*TAATACGACTCACTATAGG*GCGGCCGCACGCGTGCTGAAGTCAAGTT | 321 |
|  | Rev: C*TAATACGACTCACTATAGG*GCGGCCGCCTTTTCGTTGGGATCTTTCG |  |
| **Primers for semi-quantitative RT- PCR^4^** | | |
| *BC-Actin* | For: CTCCCTGTACGCCTCTGGT | 308 |
|  | Rev: AGCCTGGATGGCGACGTA |  |
| *BC-ATPase* | For: AAAGCCTTGAAAACTGATGTGAAA | 330 |
|  | Rev: CCTATTGACTTAAGGGCTTCGATT |  |
| *BC-Hsp70* | For: TTGCAAATGACCAAGGAAACAG | 240 |
|  | Rev: GCAGCATCTCCTATTAGACGTTCA |  |
| *BC-CLIC* | For: TTGAAGGAAGATGATGTCAGCATAA | 288 |
|  | Rev: TTCTTGCCAAGGTGGTCATTT |  |
| *BC-rRNA* | For: ACCGTCAGTTCTGCCAGTCT | 509 |
|  | Rev: TCGAGTCTCGTCCGCTTAAT |  |
| **Primers for quantitative real-time PCR^5^** | | |
| *BC-Actin* | For: AGAGAGAAGATGACCCAGATCATGT | 69 |
|  | Rev: GCAGCTCGTAGCTCTTCTCC |  |
| *BC-ATPase* | For: TTGTGCTGGACCCTACCATT | 92 |
|  | Rev: CAATCATGCCTCCAATGATG |  |
| *BC-Hsp70* | For: GACCACACCCAGTTATGTTGC | 79 |
|  | Rev: GGGAAGAAAGATTTGGTCTCG |  |
| *BC-CLIC* | For: GCTAAAAACCATTAGCTTGAAGG | 74 |
|  | Rev: TTCTTGCCAAGGTGGTCATTT |  |
| *BC-rRNA* | For: CGTGTGGAAGAACCCAAGTTC | 65 |
|  | Rev: GCCGCAAGGCAGGTTTG |  |

1 Names shown refer to the specific primer sequence indicated.

2 Nucleotide sequence (from 5’ to 3’) of specific primer is shown.

3 Italicized nucleotides are the T7 RNA polymerase promoter sequence.

4 Primers used for semi-quantitative RT-PCR in different tissues.

5 Primers were designed to amplify sequences outside of the region amplified by primers used for dsRNA synthesis.
