# Supplementary material for: Oral Delivery of Double-Stranded RNAs and siRNAs Induces RNAi Effects in the Potato/Tomato Psyllid, Bactericerca cockerelli
Source: PLoS One. 2011 Nov 16;6(11):e27736. doi: 10.1371/journal.pone.0027736 (PMC3218023; doi:10.1371/journal.pone.0027736)
Supplement: Table S2 — Annotation for the sequences used for feeding experiments in Figure S2. (DOC) [file pone.0027736.s006.doc]

**Table S2**. Annotation for the sequences used for feeding experiments in Supplementary Figure 2.

| **Contig NO.** | **Annotation** |
| --- | --- |
| 210 | ATP synthase alpha subunit |
| 698 | heat shock protein 90 |
| 813 | GTP-binding nuclear protein ran |
| 816 | ADP-ribosylation factor 1 |
| 934 | alpha-glucosidase, amylase or maltase |
| 1026 | vacuolar atp synthase subunit e |
