# Supplementary material for: Oral Delivery of Double-Stranded RNAs and siRNAs Induces RNAi Effects in the Potato/Tomato Psyllid, Bactericerca cockerelli
Source: PLoS One. 2011 Nov 16;6(11):e27736. doi: 10.1371/journal.pone.0027736 (PMC3218023; doi:10.1371/journal.pone.0027736)
Supplement: Table S3 — Quantitative real-time PCR detection for endogenous BC-ATPase mRNA after injection of dsRNAs. (DOC) [file pone.0027736.s007.doc]

**Table S3.** Quantitative real-time PCR detection for endogenous *BC-ATPase* mRNA after injection of dsRNAs^1^.

| NO. of sample^2^ | Means ± SE in GFP sample^3^ | Means ± SE in Test sample^3^ | P Value^4^ |
| --- | --- | --- | --- |
| 6 | 1 ± 0.2 | 0.73 ± 0.2 | 0.0426* |
| 7 | 1 ± 0.34 | 0.77 ± 0.25 | 0.1760 |
| 5 | 1 ± 0.18 | 0.71 ± 0.08 | 0.0114* |

1. Each individual was injected with 200nL of 100ng/µL dsRNA for *BC-ATPase* or GFP. Total RNAs from individual live psyllids were extracted two days post-injection and used for quantitative real-time PCR.
2. The number of psyllids used for qRT-PCR analysis in each experiment. For each experiment, the same numbers of GFP samples were used as controls with test sample.
3. The mRNA abundance of specific genes after introduction of corresponding dsRNA or siRNA were shown as test sample, and the average value of the control GFP group was designated as 1. Expression of each mRNA was normalized to the level of rRNA in same sample.
4. Difference between GFP group and test group was calculated and shown as P value using Bonferroni (Dunn) *t* -test. Single asterisk indicates p < 0.05.
